# The Possibility of a Central Bureau

**Published:** 1914-01-17

**Authors:** 


### HOSPITAL STATISTICS.

### The Possibility of a Central Bureau.

We recently published two articles criticising the present state of hospital statistics and making suggestions with a view to their improvement. It was pointed out that one way of meeting the really pressing requirements of the case was to be found in the establishment of a central bureau in which, with the aid of a card-index system, the principal records could be stored, sorted, and analysed. By an interesting coincidence an almost identical proposal has been made independently in the United States. We refer to a paper written by Dr. Charles F. Bolduan, assistant to the General Medical Officer of New York, and circulated by the Department of Health.*

Dr. Bolduan calls attention to the present worthlessness of hospital statistics and the need for centralisation. He proposes the use of the card system and gives a specimen of the form he recommends. This includes spaces for recording age, sex, nationality, diagnosis, length of stay in hospital, result of treatment, etc.; it is to be filled in at the hospital and forwarded to the central office. At the central bureau each certificate is examined by a trained physician-registrar who indicates on each certificate the correct designation in the standard nomenclature. The certificate is then handed over to a tabulating clerk, who transfers the information by means of punch holes to a card prepared for sorting with the Hollerith machine. This system allows of an immense variety of tabulations being made, and data so prepared could be employed in the statistical study of a host of problems with which we are at present unable to deal.

The actual details of Dr. Bolduan's system are, of course, open to criticism, but the principle is, in our judgment, absolutely sound. We understand that a strong committee of the Royal Statistical Society is at present engaged in framing proposals for the improvement of morbidity statistics. Should their final report be favourably received by the council of the Society, we trust that steps will be taken to bring it to the notice both of the individual authorities of the Metropolitan hospitals and also of the management of the King's Fund. The present state of affairs is, we think, incapable of defence, and it will be to the discredit of all concerned if reforms are not speedily introduced.

* *Hospital Morbidity Statistics: A Practicable Method of Making them Uniform and Preparing them for Analytic Study.* By Charles F. Bolduan, M.D. Pp. 11. (Department of Health of the City of New York. Reprint Series No. 5. April 1913.)

